# Has the Polyploid Wave Ebbed?

**DOI:** 10.3389/fpls.2020.00251

**Published:** 2020-03-10

**Authors:** Donald A. Levin

**Affiliations:** Department of Integrative Biology, University of Texas at Austin, Austin, TX, United States

**Keywords:** divergent evolution, diversification lag, dysploidy, extinction, polyploidy, reproductive isolation

## Abstract

There was a wave of whole genome duplications (WGD) during and subsequent to the K-Pg interface, which was followed by an increase in the proportion of species that were polyploid. I consider why this wave of polyploid speciation has continued to rise through the divergent evolution of polyploid lineages, and through rounds of homoploid and heteroploid chromosomal change. I also consider why the polyploid speciation wave is likely to rise in the next millennium. I propose that the speed of polyploid genesis through ploidal increase and through diversification among polyploids likely will be greater than the speed of diploid speciation. The increase in polyploid diversity is expected to lag well behind episodes of WGD, owing to the very long period required for species diversification either by lineage splitting or additional rounds of polyploidy, in addition to the long period of genomic adjustment to higher ploidal levels in neopolyploids.

## Introduction

The K-Pg interface was a period of intense global change associated with the mass extinction event that ended the Age of Dinosaurs (Nichols and Johnson, 2002). Terrestrial plant ecosystems were altered dramatically at the K-Pg interface 65.5 million years ago (Ma), in part because of the massive extinction of species, genera, and higher lineages (Wolfe and Upchurch, 1986; Vajda et al., 2001; Nichols and Johnson, 2002; McElwain and Punyasena, 2007). For example, paleobotanical studies of fossil leaves, pollen and spores from North American sites have shown that 18–30% of plant genera and up to 80% of all plant species died out during the K-Pg interface (Nichols and Johnson, 2002; Wilf and Johnson, 2004). Jablonski (1994) estimated that 47% of all marine genera died out during this period, as did 76% of all species.

Evolutionary rebounds following mass extinctions are important components of micro- and macroevolution (Jablonski, 1986; Benton, 1987). The recovery of biodiversity subsequent to mass extinctions is rapid by geological time scales, and frequently is coincident with significantly accelerated evolutionary rates (Miller and Sepkoski, 1988). The period during and subsequent to the K-Pg boundary was a particularly active one for plant polyploidizations, as there was a wave of whole genome duplications (WGD), i.e., a clustering of plant polyploidizations, during this time (Fawcett et al., 2009; Vanneste et al., 2014; Cannon et al., 2015). Many WGDs in different plant families occurred roughly between 60 and 65 million years ago (Van de Peer et al., 2017; Clark and Donoghue, 2018). The numerous independent WGD events across multiple angiosperm lineages may be correlated with major climatic perturbations, and are clustered at the base of some of the most successful and largest extant plant families and larger clades (Soltis et al., 2015). For example, Cai et al. (2019) found a significant correlation between the timing of WGD events in the Malpighiales and periods of global climatic change during the Paleocene–Eocene, ca. 56–54 million years ago.

Multiple adaptive WGDs have occurred in the history of very successful clades. For example, there have been 13 independent polyploid events in the Brassicaceae (Mandáková et al., 2017); and 43% of the species therein are neopolyploids (Hohmann et al., 2015). There have been 26 ancient and more recent polyploidy events in Caryophyllales (Yang et al., 2017); and numerous polyploidization events have occurred throughout the Asteraceae (Huang et al., 2016). Multiple, adaptive shifts involving physiological, morphological and/or ecological attributes have occurred in the annals of successful phylads. These shifts may be the immediate product of ploidal change, and/or they may be the result of selective responses within polyploid species. The more the environment changes, the more varied the types and the magnitude of adaptive shifts are likely to be. Changes in the physiological, morphological and/or ecological attributes within single phylads need not be concurrent, nor will a given type of change necessarily occur synchronously across phylads inhabiting the same environments.

## The Premise

If many WGDs in different plant families occurred within 10 million years or so after the K-Pg interface, and if there was a polyploid wave that commenced at that time, we may ask whether the polyploid wave has ebbed, and what the wave is likely to do in the future. I argue here that the polyploid wave has been growing since its inception, and that is likely to continue to do so.

Although WGD changes near the K-Pg boundary have received the most attention, there have been numerous pulses of polyploidy during the past 40 million years within several families. For example, repeated evolution of polyploids in the grass tribe Andropogoneae occurred during the expansion of C4 grasslands in the Late to Mid Miocene (Estep et al., 2014). A surge of polyploidy occurred in several tribes of the Asteraceae in the Early Middle Miocene (Huang et al., 2016), and in the Brassicaceae in the mid to late Miocene (Kagale et al., 2014). Jacob Landis (personal communication) estimated that 7 WGD events associated with diversification pulses occurred between 33 and 37 mya, and 6 such events between 12 and19 mya, compared to 14 WGD events between 50 and 60 mya.

Polyploidy has been an ongoing process. Some polyploids are roughly a million years old, as are allotetraploid *Melampodium strigosum* and two allohexaploids (McCann et al., 2018). Other polyploids have emerged within the past 25,000 years. For example, the allotetraploid *Arabidopsis kamchatica* has evolved around 20,000 years ago (Novikova et al., 2018). The tetraploid *A*. *arenosa* arose from one population about 15,000–19,000 years ago; *A. suecica* is about 16,000 years ago. Polyploid species also have arisen within the past 300 years. They include *Spartina anglica, Senecio squalidus, Senecio eboracensis, Senecio cambrensis, Mimulus peregrinus* (Thomas, 2015). *Tragopogon mirus* and *T. miscellus* are only about 100 years old (Soltis and Soltis, 2009). Some recent polyploids may prove to be the antecedents of a speciation surge within a genus or family; it depends on their persistence and rate of diversification.

Polyploid waves take millions of years to develop. Within a family or higher phylad, polyploidy originates within one genus, and then perhaps many others; and only much later polyploidy may become prominent. This process would be reflected in phylogenetic trees, where entire branches or groups of twigs will be polyploid.

As briefly summarized above, there is considerable documentation of the rise of polyploids subsequent to K-Pg interval, and their continued production from diploid progenitors. However, there are some aspects of the polyploid scenario which have received relatively little attention; and their consideration would expand our understanding of the polyploid dynamic. This paper considers three aspects, namely (Nichols and Johnson, 2002) mechanisms of polyploid diversification, Wolfe and Upchurch (1986) reasons why WGD are not followed by immediate diversification thrusts, and (Vajda et al., 2001) polyploidy going forward.

## Mechanisms of Polyploid Diversification

Whereas the rapid transition from diploidy to tetraploidy has been the focal point in discussions of polyploid increase, ostensibly most polyploid species do not arise directly from diploid progenitors. There are two mechanisms which contribute most to an increase in the percentage of polyploid species. They are additional rounds of polyploidy, and divergence within polyploid lineages. Polyploid species can produce only more polyploids. They cannot beget diploid species, whereas diploid species can produce both diploid and polyploid species (Meyers and Levin, 2006). The percentage of speciation events that contributed to the origins of other polyploids through additional rounds of polyploidy or via divergent processes remains to be determined.

Consider first additional rounds of polyploidy. Hybridization between a tetraploid species and a diploid can yield sterile triploid hybrids, whose unreduced triploid gametes may fuse to produce fertile hexaploids (Ramsey and Schemske, 1998). Or sterile tetraploids from hybridization between chromosomally differentiated tetraploid species can produce unreduced tetraploid gametes which may fuse to produce fertile octaploid individuals. Some very high ploidal levels have been reached in genera such as *Viola*, in which *V. arborescens* is at least 20-ploid (2n = ca. 160), and two lineages are 14-ploid and one is 18-ploid lineage (Marcussen et al., 2012). Some genera are composed primarily of polyploid species [e.g., *Triticum* and *Festuca* (70%), and *Draba* (78%)] (Soltis et al., 2015). In old species complexes, members with lower ploidal levels may be absent. For example, in *Bromus* section Ceratochloa there are no diploid or tetraploid species, but there are 8-ploid and 12-ploid species (Grant, 1982). A shift from a predominance of diploid species in a species complex to one where polyploids prevail also may be indicative of polyploid superiority.

Polyploid species also may generate other such species via divergent evolution. Allopatric and peripatric speciation may occur within tetraploid or hexaploid genera (for example) just as it does within diploid genera. Intraspecific lineages gradually diverge and accumulate genetic differences which confer ecological isolation or which result in the emergence of post-pollination barriers such as cross-incompatibility, hybrid inviability, and hybrid sterility. What we know about divergent evolution in diploid species via genetic change in response to disparate environmental challenges or opportunities also pertains to polyploids. The tempo of barrier building in polyploid lineages through genetic change alone is not well understood. In diploid lineages, strong ecological barriers typically emerge only after tens if not hundreds of thousands of years; and cross-incompatibility and hybrid sterility typically arise only after many millions of years (Levin, 2012, 2013).

The strength of post-zygotic isolation may be lower between polyploids than between diploids and polyploids (Hersch-Green, 2012; Hülber et al., 2015; Sutherland and Galloway, 2017). For example, in *Senecio carniolicus*, plants with intermediate ploidal levels were absent in diploid/tetraploid and diploid/hexaploid contact zones, but were frequent in a tetraploid/hexaploid zone (Hülber et al., 2015). In the *Aster occidentalis* complex, viable hybrids were obtained from 26% of the crosses between diploid species versus 66% of the crosses between polyploids (Allen et al., 1983). Tetraploid/hexaploid hybrids within *Jacobaea carnifolica* had greater seed germination and seedling viability than diploid/polyploid hybrids within the species (Sonnleitner et al., 2013). High ploidal entities may act as evolutionary “sponges,” expanding their base of variation through introgression across or within ploidal levels (Baduel et al., 2018).

Speciation among polyploids also may involve chromosomal change without a shift in ploidal level (Leitch and Bennett, 2004; Leitch and Leitch, 2008). The fixation of chromosomal novelty most often will be associated with migration and population bottlenecks. Fixation involves stochastic processes, wherein the greater the bottleneck or the fewer the number of founders of new chromosomal populations, the lower is the fixation probability (Lande, 1985; Rieseberg, 2001; Jackson et al., 2016). Correlatively, the greater the level of relatedness among survivors of bottlenecks, the higher the likelihood that a new chromosomal arrangement will be fixed (Wright, 1969; Hedrick and Levin, 1984). The species prone to chromosome breakage could experience a series of novel rearrangements in one population that is unlikely to arise elsewhere. Moreover, their fixation would be associated with loss of genetic diversity, which would be exacerbated by subsequent migration/colonization bottlenecks (Excoffier et al., 2009). Accordingly, chromosomally differentiated lineages/species are likely to be narrowly distributed, short-lived evolutionary entities; and the new karyotype likely would have a narrow geographical footprint.

Past polyploidizations often have been followed by additions or subtractions in chromosome number within a ploidal level, i.e., dysploidy (Mandáková et al., 2017; Mandáková and Lysak, 2018). Descending dysploidy relies on translocations among homoelogous chromosomes and/or non-homologous chromosomes. Ascending dysploid based on centric fusions also is possible (e.g., in palms, Barrett et al., 2019), but it is less frequent than the descending mode. Dysploidy is one of the most important avenues to diploidization. Progressive diploidization may reduce chromosome numbers, such that the chromosome number of a species with a WGD in its history may be the same as relatives without such (e.g., *n* = 10 in maize with a WGD and in sorghum without WGD, 50). The extent and rate of dysploidy is expected to be positively correlated with ploidal level and the time since a ploidal increase (Mandáková and Lysak, 2018). Accordingly, ancient hexaploids should have higher levels of dysploidy than tetraploids. Indeed, this relationship was found across several clades in the Brassicaceae (Mandá ková et al., 2017).

If dysploidy is associated with polyploidy, it follows that there would a secondary wave of dysploidy lagging behind the post K-Pg polyploid wave. The magnitude of the dysploid wave remains to be determined. Since dysploid species may serve as platforms for additional rounds of polyploidy (Wendel, 2015), their presence likely added impetus to the polyploid wave. The dysploid wave actually may be much higher than the polyploid wave. Whereas the number of ploidal jumps that genera are likely to accommodate are limited, there could be well over ten euploid number shifts within a genus (Mandá ková et al., 2017; Mandáková and Lysak, 2018).

## Why WGD Are Not Followed by Immediate Diversification Thrusts

Ren et al. (2018) found that lineages with early WGD such as Brassicaceae Malvaceae, Fabaceae, Asteraceae and Poaceae have significantly greater species diversity than their sister lineages. They also detected that species radiation was higher in orders with a greater percentage with WGDs than smaller orders for Asparagales, core Lamiales, and core monocots. Correlatively, in *Allium* higher species diversification rates occurred in lineages with high polyploid frequencies (Han et al., 2020). Whether polyploidy is a universal promoter of species diversification remains to be determined.

If polyploidy promotes species diversification, there are several reasons why that boost probably would not be immediate. It has been proposed that diversification via ploidal change requires genome stabilization through diploidization, the genesis of novel key traits, and the buildup of genetic diversity; and these processes take a millions of years (Landis et al., 2018). The pace of diploidization varies among phylads and components therein, as does the marshaling of genetic diversity. Once the genome has settled and variation has accrued, speciation may proceed through the evolution of ecologically and geographically distinctive intraspecific lineages. Subsequent genetic and/or chromosomal divergence would lead to their reproductive isolation (Clausen, 1951; Levin, 2000). Even additional rounds of polyploidy would take a long time to have a substantive effect on diversity, because neopolyploids are likely to be short-lived, and because the products of one episode of ploidal increase may be similar to those of another (Levin and Soltis, 2018). The components of some species may exploit similar types of habitats due to genetic constraints and poor niche availability or opportunities. As such, their subsequent rate of radiation will be retarded relative to more ecologically flexible species. These species most likely will originate from multiple origins in which the parental entities were adapted to somewhat different habitats. Finally, the breeding system may contribute to the diversification lag in some clades. Families that are exclusively self-compatible have lower net diversification rates than those that are predominantly self-incompatible (Ferrer and Good, 2012). Ostensibly selfing species having higher extinction rates than outcrossers (Wright et al., 2013) in part due to a reduced ability to adapt, especially to new environments (Hartfield et al., 2017).

The absence of immediate speciation acceleration after polyploidy also may be the product of species extinction, because diversification is measured as speciation minus extinction. Consider first a tree (phylogenetic or otherwise) with a single stem. Branching into two axes may subsequently occur, and each branch (A and B) may bear additional branches, and so forth. However, for tree complexity to increase, each new branch must remain on the tree. Branch loss will delay or prevent subsequent tree radiation. The greater the incidence of branch loss the longer the time for a complex tree to develop. It follows that species extinction would thwart diversification. The establishment of a polyploid genus may be followed by long intervals in which only one or a few species remain viable. Then the subsequent generation of diversity would lag well behind the establishment of the first polyploid(s). If a genus radiated from an original polyploid, and then experienced a major and prolonged contraction, a subsequent expansion would lag behind the origin of the first polyploid.

The likelihood of polyploid species extinction is an inverse function of the number of ecogeographically differentiated lineages within a given species (Rosenblum et al., 2012). Divergent gene pools offer alternate points of departure for selective responses to different abiotic and biotic challenges, thus improving chances of local survival or migration in the face of environmental change (Oney et al., 2013; Moran et al., 2016). This is because ecogeographically differentiated lineages are unlikely to have similar geographical footprints. Indeed these lineages may live in different areas; and they are unlikely to respond to environmental change in the same manner or degree (Bennett and Provan, 2008; Stewart, 2009; Stewart et al., 2010). Polyploid species with many highly divergent lineages are likely to have broader ranges than species with few such lineages (Gaston and Fuller, 2009; Morin and Lechowicz, 2013; Slatyer et al., 2013). Range size tends to be positively correlated with species longevity (McKinney, 1997; Saupe et al., 2015).

## Polyploidy Going Forward

Given that the percentage of polyploids has been increasing, what might be expected of the contribution of polyploids to future flowering plant diversity? I propose that the percentage of species that are polyploids almost certainly will increase during the next several millennium. If 25 to 30% of extant flowering plants are recent polyploids (Wood et al., 2009; Barker et al., 2016), then several millennia from now it would not be surprising to see that number approach 35–40%. A significant increase is likely because the factors that contributed to the Anthropocene polyploid formation such as habitat disturbance, transport, and domestication (Thomas, 2015; Bull and Maron, 2016; Vellend et al., 2017; Otto, 2018) are likely to have a larger impact in the future. Moreover climate change will alter community structure and create new habitats in which polyploids may thrive (Vanneste et al., 2014; Van de Peer et al., 2017). Hundreds of species in various parts of the world have already become locally extinct (Wiens, 2016). The background rate of extinction is 1000–10,000 times higher than it has ever been (Pimm and Joppa, 2015). The extent to which polyploids have outlived diploids over the past 5000 years remains to be determined.

Polyploidy can produce the antecedents of new species in one generation (autopolyploidy) or two generations (allopolyploidy) through the production of unreduced gametes or doubled somatic cells (Ramsey and Schemske, 1998; Mason and Pires, 2015). Autopolyploids can arise anywhere in a species’ range from single individuals via the formation of unreduced gametes. Because autopolyploid genesis bypasses the requirement for species contact and hybridization, autopolyploids almost certainly will evolve much more frequently than allopolyploids. This is not to suggest that over the longer term autopolyploid species will prevail, which they do not (Barker et al., 2016). Allopolyploids tend to have broader niche tolerances than autopolyploids, and to be more ecologically divergent from their progenitors than are autopolyploids (Levin, 2002).

Speciation among polyploids also may involve chromosomal change without a shift in ploidal level. The fixation of chromosomal novelty and dysploid numbers most often will be associated with migration and population bottlenecks. Such change may occur in only a few hundred generations or less; and this form of speciation may be more important than a change in ploidal level. New polyploids also may originate from existing polyploids via the divergence of gene pools without chromosomal change. This is likely to be the least important speciation mode over the next few millennia, because ecological and incompatibility barriers take very long periods to evolve, as discussed above. It is possible, however, that some divergent lineages within species will become reproductively isolated within the next few thousand years.

A persistence/extinction differential between diploid and polyploid species would have an impact on the ploidal balance. If polyploid species were more tolerant of the upcoming climatic changes than diploids, as they seem to be and have been (Fawcett et al., 2009), then polyploid representation would increase. It is likely that the climatic shocks near and following the K-Pg boundary contributed to a diploid survival trough, and to a decline in the proportion of diploid species (Vanneste et al., 2014; Van de Peer et al., 2017; Levin and Soltis, 2018). If polyploids increase proportionally, then it will be most pronounced in herbaceous species, whose rates of chromosome doubling were six times higher than in woody species (Zenil-Ferguson et al., 2017). A polyploid elevation also is likely to be more pronounced in perennial herbs than in annuals (Otto and Whitton, 2000).

If polyploids had a higher survival rate than diploids over the past 5000 years, then we would surmise that the diploid:polyploid balance would be tilted toward the latter in the future. Unfortunately past survival rates are unknowable. However, an insight into a possible differential may be obtained from a comparison of the ploidal levels of endangered species and their invasive relatives. Endangered plants are more likely to be diploid than their invasive congeners or invasive species as a whole (Pandit, 2006; Pandit et al., 2011). Pandit et al. (2011) found that being endangered is 14% more likely for diploids than for polyploids. The bases for this differential remains to be determined. Polyploids might benefit from the immediate effects of ploidal increase alone, or through a higher level of genetic diversity, or a combination of both. Perhaps polyploids will have a higher survival rate than they did during preceding periods.

## Conclusion

The polyploid wave which began roughly 60 mya continues to rise. Pulses of polyploidy have occurred many times since then. Roughly 30% of all flowering plants are polyploid; and this number is likely to increase in the coming several millennia, perhaps approaching 40%. The factors promoting polyploidy in the past surely will be important in the future. These factors include climate and environmental change, which lead to community disassembly, and migration and contact between previously isolated congeners. Anthropogenic drivers will have an increasing impact going forward. In addition to elevated polyploid production, the percentage of polyploid species is likely to increase because diploid species may be more vulnerable to environmental change than polyploids.

The increase in polyploidy in the coming millennia will involve not only the doubling of diploid chromosome numbers, but also the genesis of higher level polyploids and dysploids from existing polyploids. A wave of dysploidy almost certainly accompanied the polyploid wave of the past, because polyploids are much more prone to chromosomal change than diploids, and because one dysploid species could parent another with a different chromosome number. Divergent evolution within a polyploid or dysploid species also may propel the genesis of more polyploids. However, that process is slow, and thus unlikely to yield large numbers of species within a few thousand years.

Some products of ploidal shifts several million years ago were the antecedents of major lineages/clades. However, diversification in the latter lagged well the WGD themselves, because new polyploids tend to have high extinction rates regardless of mode of origin, and because diversification in its various forms takes a very long time. Accordingly, new polyploid species that evolve within the next few millennia are unlikely to parent many species by the end of that period. Over millions of years, however, some may prove to be the antecedents of major lineages/clades.

## Author Contributions

The author confirms being the sole contributor of this work and has approved it for publication.

## Conflict of Interest

The author declares that the research was conducted in the absence of any commercial or financial relationships that could be construed as a potential conflict of interest.
